# Collargol in Puerperal Fever

**Published:** 1909-08

**Authors:** 


COLLARGOL IN PUERPERAL FEVER.

Pinel (*Centralbl. f. Gynak.*, 1905, No. 25) says that the therapeutic effect of collargol in puerperal sepsis is indisputable. Collargol acts first on the general condition and then reduces temperature and pulse. But the other therapeutic measures should not be neglected.

Hoummel (*Presse médicale*, 1906, No. 2) described a puerperal infection in which all treatment was without avail, so that the temperature rose to 40° C. (104° F.) and maintained this height for two days. Experimentally he injected 60 minims of a 1 per cent. collargol solution into the womb and tamponaded the cervix, so that the solution was retained. Twelve hours later the temperature had sunk to 37° C. (98. 6° F.) and the general condition rapidly improved; the patient got out of bed eight days later.

Osterloh (*Jahresber. d. Gesellsch. f. Natur- u. Heilkunde*, 1904-5) reviews the collargol literature and reports a case of puerperal fever in a primapara after manual removal of placenta. Thrombosed vessels in both parametria, daily chills. Treatment: four intravenous injections of each 7 to 8 c.c. of 2 per cent collargol, at intervals of two days, and twelve enemata of each 50 c.c. of 1 per cent. collargol, at intervals of two days. After five days no more chills. The effect of collargol was favorable on the chills and the subjective condition. He considers collargol especially indicated in general puerperal sepsis without localisation.

Keim (*Les Medications Nouvelle en Obstetrique;* Balliere, Paris, 1908), recommends collargol in septic thrombophlebitis and ophthalmia neonatorum.

Kraus (*Centralbl. f. Gynäk.*, No. 40, 1906,) advises in acute peritoneal processes post partum and abortum the rectal injection of 50 c.c. collargol solution (1 per cent.) twice daily and a tablespoonful by mouth hourly. In six cases he saw as effect disappearance of the inflammatory peritoneal symptoms and of intestinal paralysis, as well as passage of great amounts of flatus and of thin, blackish fecal masses.

Williams (*Practical Gynecology;* F. A. Davis Co., Philadelphia, 1908) writes under tuberculous endometritis) : The silver treatment is on trial, with much to sustain its claim as a germicide and antitoxic. It has been found that germs will not propagate in the vicinity of silver and that suppuration seldom occurs in the track of silver sutures. The preparation used is known as collargol, an allotropic form of pure silver. This is absolutely nontoxic in any quantity and argyria has never been known to follow its use. It may be used per os in pill or solution, by intravenous injection, or in the form of an ointment applied to an absorbable surface. This latter is the form in which it is habitually used by the originator. Credé's ointment contains 15 per cent. collargol. The surface to which it is to be applied—the inner surface of the thighs or other portion of the body where the skin is soft and thin—is to be cleaned and the ointment rubbed in for from 20 to 30 minutes or until it has all disappeared. The quantity used is usually from 15 to 45 grains, but there need not be any limit. The applications may be repeated from one to half a dozen times in the twenty-four hours.

Further publications favorably commenting on the use of collargol in the treatment of puerperal fevers have been rendered by Weindler (*Centralbl. f. Gynäk.,* 1905, No. 13) ; Rosenstein (*Monatsschr. f. Geb.,* Vol. XVII, 3|4) ; Aronsohn (*Therapie d. Gegenw.,* March, 1908) ; and many others.

